# Malignant myoepithelioma of the hard palate: 9-year follow-up

**DOI:** 10.1016/S1808-8694(15)30506-1

**Published:** 2015-10-19

**Authors:** Lucas Gomes Patrocinio, Priscila Garcia Damasceno, José Antonio Patrocinio

**Affiliations:** 1 Otolaryngologist and Craniofacial Surgeon, Head of the Craniofacial Division of the Otorhinolaryngology Ward - Medical School of the Federal University of Uberlândia; 2 ENT resident - Otorhinolaryngology Ward - Medical School of the Federal University of Uberlândia; 3 Full Professor - Chairman - Otorhinolaryngology Ward - Medical School of the Federal University of Uberlândia. Serviço de Otorrinolaringologia da Faculdade de Medicina da Universidade Federal de Uberlândia, Uberlândia, Minas Gerais, Brasil

**Keywords:** myoepithelioma, head and neck neoplasms, hard palate

## INTRODUCTION

Myoepitheliomas are rare tumors that represent about 1% of the salivary gland tumors^1^. Most of them are benign, and only 10% are malignant, and the latter are called malignant myoepitheliomas or myoepithelial carcinomas^1^. The first case of a malignant myoepithelioma was described in 1975, since then there has been a greater incidence of these tumors reported in the parotid gland^1^. Its involvement of the hard palate is extremely rare, and there are only 8 cases reported in the world literature and with short term follow up^1^, ^2^, ^3^, ^4^, ^5^, ^6^.

The present investigation reports a case of a patient with a malignant myoepithelioma on the hard palate, with bone destruction, successfully operated upon.

## CASE REPORT

**Figure 1:**
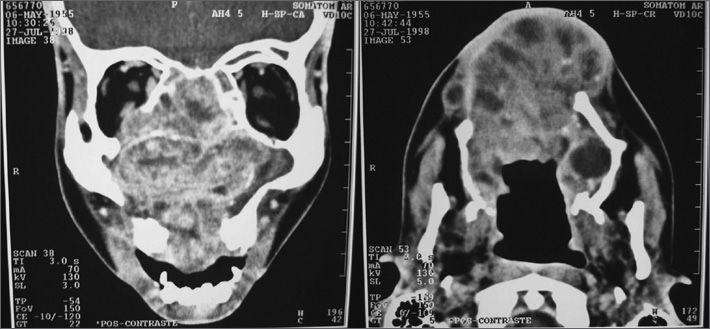
CT scan showing a large solid mass occupying part of the right-side maxillary sinus, palate and nasal cavity.

R.A., male, 38 years old, complaining of nasal obstruction for years, associated with running nose and recurrent epistaxis. During exam we noticed a palate tumor extending to the right-side nasal cavity. Computerized tomography (CT) showed a large solid mass occupying part of the right maxillary sinus, palate and nasal cavity (Fig. 1).

He was submitted to a transoral resection of the tumor, which pathology exam showed a tissue neoformation made up of ovoid cells of clear cytoplasm with round nuclei and, sometimes, spindle-shaped cells with areas of stromal hyalinization and cystic formations. Immunohistochemistry analysis was positive for 14 cytokeratin, vimentin and specific muscle actin, which result matches the description of a malignant myoepithelioma. He had two new recurrences, also treated surgically.

We made him a palate closure prosthesis as a means for functional reconstruction. He has been under follow up for nine years, without signs of recurrence.

## DISCUSSION

Malignant myoepitheliomas are rare tumors made up of atypical myoepithelial cells with high mitotic activity and aggressive growth1. Such tumors may stem from the differentiation of a benign tumor, it can stem from a benign tumor or it may recur, which is the most frequent situation^2^, ^3^.

The parotid gland is the most common tumor location, followed by the palate and the submandibular gland^1^. There is no gender predominance and the mean age is 62 years^1^. It is usually painless, which delays diagnosis1. Malignant myoepitheliomas are characterized by local invasion and destruction, and it rarely metastasizes, and when they do, they involve lungs, liver, bones and lymphnodes^4^.

Histologically, the malignant myoepithelioma is characterized by pleomorphism, occasionally with eosinophilic cytoplasm, a high mitotic rate and usually with necrosis^5^, ^6^. There are many architectural patterns (solid, myxoid and reticular) and different cell types: spindle, epithelioid, plasmocytoids and clear cells^5^, ^6^.

Differential diagnosis includes leiomyosarcoma, peripheral nerve sheath nerve tumor, synovial sarcoma and metastatic melanoma, and immunohistochemistry is fundamental do differentiate them5. It shows constant positiveness for the S100 protein, vimentin and cytokeratin antibodies^3^. Cytokeratin expression is variable in spindle-cell tumors^3^. The specific muscle actin immunoreaction varies according to cell phenotype^3^.

The treatment advocated is tumor surgical resection with margins; however, before such procedure, an image exam must be carried out in order to assess the extension and involvement of neighboring structures^1^, ^2^. In the literature studied, all the cases were treated by surgical resection, and the outcomes were favorable.

## CONCLUSIONS

The malignant soft palate myoepithelioma is an extremely hard tumor. Its treatment continues being broad resection. The long patient follow up described in the present case corroborates literature data.

## References

[1] Nagao T, Sugano I, Ishida Y, Tajima Y, Matsuzaki O, Konno A, Kondo Y, Nagao K (1998). Salivary gland malignant myoepithelioma: a clinicopathologic and immunohistochemical study of ten cases. Cancer..

[2] Karatzanis AD, Drivas EI, Giannikaki ES, Lachanas VA, Hatziioannou JK, Velegrakis GA (2005). Malignant myoepithelioma arising from recurrent pleomorphic adenoma of the soft palate. Auris Nasus Larynx..

[3] Bombi JA, Alos L, Rey MJ, Mallofre C, Cuchi A, Trasserra J, Cardesa A (1996). Myoepithelial carcinoma arising in a benign myoepithelioma: immunohistochemical, ultrastructural, and flow-cytometrical study. Ultrastruct Pathol..

[4] Chhieng DC, Paulino AF (2002). Cytology of myoepithelial carcinoma of the salivary gland. Cancer..

[5] Kuwabara H, Uda H, Miyabe K, Saito K, Shibanushi T (1998). Malignant plasmacytoid myoepithelioma of the palate: histological observations compared to benign predominant plasmacytoid myoepithelial cells in pleomorphic adenoma of the palate. Ultrastruct Pathol..

[6] Kuwabara H, Kohno K, Kishida F, Uda H, Miyabe K, Nagao K, Saito K, Shibanushi T (1998). Imprint cytology of malignant plasmacytoid myoepithelioma of the palate. Acta Cytol..

